# Generative Models for Periodicity Detection in Noisy Signals

**DOI:** 10.3390/clockssleep6030025

**Published:** 2024-07-23

**Authors:** Ezekiel Barnett, Olga Kaiser, Jonathan Masci, Ernst C. Wit, Stephany Fulda

**Affiliations:** 1 NNAISENSE, 6900 Lugano, Switzerland; ezekbarnett@gmail.com (E.B.); olga.kaiser@nnaisense.com (O.K.); jonathan.masci@nnaisense.com (J.M.); 2 Institute of Computing, Università della Svizzera Italiana, 6962 Lugano, Switzerland; wite@usi.ch; 3 Sleep Medicine Unit, Neurocenter of Southern Switzerland, EOC, 6900 Lugano, Switzerland

**Keywords:** periodicity, periodicity detection, algorithm, generative models, periodic leg movements during sleep

## Abstract

We present the Gaussian Mixture Periodicity Detection Algorithm (GMPDA), a novel method for detecting periodicity in the binary time series of event onsets. The GMPDA addresses the periodicity detection problem by inferring parameters of a generative model. We introduce two models, the Clock Model and the Random Walk Model, which describe distinct periodic phenomena and provide a comprehensive generative framework. The GMPDA demonstrates robust performance in test cases involving single and multiple periodicities, as well as varying noise levels. Additionally, we evaluate the GMPDA on real-world data from recorded leg movements during sleep, where it successfully identifies expected periodicities despite high noise levels. The primary contributions of this paper include the development of two new models for generating periodic event behavior and the GMPDA, which exhibits high accuracy in detecting multiple periodicities even in noisy environments.

## 1. Introduction

From heartbeats to commutes, global climatic oscillations to Facebook log-ons, periodicity—the phenomenon where events occur at regular intervals—is ubiquitous. Detecting periodicity in time series, often referred to as the “periodicity detection problem”, is crucial across various fields. In binary time series, which indicate only the occurrence of events, this problem has traditionally been tackled using algorithms such as Fast Fourier Transform (FFT) and autocorrelation, typically focusing on single, stationary periodicities [1,2,3,4,5]. However, several challenges remain inadequately addressed in the literature, including (i) the development of generative models that accurately describe noise in periodic behavior, considering variance in interval length and false positives and negatives; (ii) the detection of multiple overlapping periods; and (iii) non-stationary periodic signals.

Existing periodicity detection algorithms, primarily based on FFT or the Autocorrelation Function (ACF), often focus on single-period detection. FFT maps a time series to the frequency domain, using the inverse of the frequency with the strongest power as the predicted period. However, FFT is sensitive to sparse data [6], noise [7], and suffers from “spectral leakage” at low frequencies/large periods [8]. The Lomb–Scargle periodogram [7,9], a least-squares method for fitting sinusoids, also shares these problems and may not be suitable for non-stationary signals. Furthermore, in real applications, the hierarchy implied by the FFT may not be appropriate to describe the signal, especially when the periodic signals are random walks with Markov properties and the signal is non-stationary.

ACF-based methods estimate similarity between sub-sequences of event intervals, selecting periods that maximize the ACF. These methods have been used for multiple periodicity detection in character series such as texts [10,11]. However, ACF detects numerous candidate periods, often requiring a self-selected significance threshold to identify true periodicities, and struggles with smaller data sets. These methods are generally not designed for multiple periodicities in event time series.

Alternative approaches include E-periodicity [12], which focuses on single period detection using the modulus operation for unevenly/under-sampled time series. E-periodicity segments the time series into all possible periodicities within some a priori specified range. It then overlays the segments and selects the true periodicity as the periodicity that ”covers” the most events. Methods like partial periodic patterns, chi-squared tests [13], max sub-pattern trees [13], and projection-based techniques [14] also target single periodic patterns in stationary signals and face challenges with low-frequency periodicities and low sampling rates [15].

Multiple period detection in time series event data has received limited attention. Most methods use a hierarchical extraction approach, iteratively removing the frequency with the highest power (FFT) or the most probable periodicity (ACF). While FFT is suitable for complex functions, it is not designed for event data [16]. ACF-based methods, combined with comb filters, identify periods but lack robustness against noise [17].

Generative models, like those in [18], describe discrete signals with Gaussian PDFs for periods and Poisson processes for noise but fail to detect multiple overlapping periods. Other approaches in “periodic pattern-finding” identify time slots for periodic events, optimizing for anomaly detection tasks with low sampling rates [15].

To address some of the challenges of multiple periodicity detection for noisy event time series, we propose the Gaussian Mixture Periodicity Detection Algorithm (GMPDA). This algorithm is based on a novel generative model scheme for periodic event time series, incorporating Gaussian-distributed noise to account for interval variability. We compare the GMPDA to existing algorithms across extensive test cases, demonstrating superior performance in accuracy, sensitivity, and computational efficiency.

The rest of the paper is organized as follows. In Section 2, we introduce the generative models and discuss their inference. Section 3 presents the GMPDA. The performance of the GMPDA framework is tested in Section 4. An application of the GMPDA to real data is detailed in Section 5. Section 6 concludes this paper.

## 2. Generative Models

Consider a uni-variate event time series ${\left(X_{t}\right)}_{t=1,\cdots ,N_{T}}$, where $x_{t}=1$ if there is an occurrence of an event at time *t*, and $x_{t}=0$ otherwise. In this work, we disregard cases of unsampled or missing data. The information in $X_{t}$ can be condensed into the set of non-zero time stamps $S:={\left\{s_{i}|x_{s_{i}}=1\right\}}_{i=1,\cdots ,N_{S}}$.

If the positive time stamps occur at regular intervals, the time series exhibits periodic behavior, and these intervals correspond to periodicities or periods. We frame the periodicity detection problem as the search for the set of periodicities that explain the intervals between time stamps in *S*.

We are particularly interested in the set of *prime* periodicities, defined as the smallest integer frequency that describes the intervals. For instance, for a time stamp set S={12,23,34,45,56,67}, several intervals could be explained by a periodicity of 22 or 33 but 11 would be the prime period, as it explains the data best, and 22 and 33 are integer multiples of this prime period. The set of underlying prime periods in $X_{t}$ is denoted by $\mu^{*}=\left\{\mu_{p}^{*},p=1,\cdots ,P\right\}$. Additionally, we assume that for most real-world applications, the interval between two consecutive time stamps associated with a periodicity $\mu_{p}^{*}$ in *S* will typically vary around $\mu_{p}^{*}$ with a variance denoted by $\sigma_{p}^{*2}$, i.e., $s_{i+1}^{p}-s_{i}^{p}\in \left[\mu_{p}^{*}-\sigma_{p}^{*},\mu_{p}^{*}+\sigma_{p}^{*}\right]$.

If the time series $X_{t}$ is generated by a single, stationary periodicity $\mu_{1}^{*}$, we can compute $\mu_{1}^{*}$, and thus the prime periodicity $\mu^{*}$, directly from the data as:

(1) $$\mu^{*}=\frac{N_{T}}{\left|S\right|}=\frac{\sum_{i=1}^{N_{T}}s_{i+1}-s_{i}}{\left|S\right|}.$$

The first equality in (1) represents the ratio between the length of the time series and total number of events. The second equality in (1) describes the “average interval” between two adjacent time stamps and holds for a time series with a single, stationary periodicity without noise. The associated variance $\sigma^{*2}$ can be estimated as the square of the standard deviation.

However, estimation of $\mu^{*}$ and $\sigma^{*2}$ using Equation (1) is insufficient when (i) the time series $X_{t}$ is generated by multiple, overlapping periodicities, (ii) the time series $X_{t}$ is noisy (i.e., contains false positives), (iii) there are missing values (false negatives), or (iv) there are varying patterns of periodic behavior over time (non-stationarity).

Our generative model addresses challenges (i) and (ii). Specifically, we formulate a generative model of the positive time stamps *S* with multiple periodicities, incorporating an explicit noise term and a loss function that enables inference of the model parameters.

Assume the set of positive time stamps *S* can be generated by a function *f* as:

(2) $$S=f(\mu^{*},\sigma^{*},\beta ,\alpha ,M),$$

where we have the following:

- $\mu^{*}$ is the set of *P* prime periodicities in the time series;
- $\sigma^{*}$ is the set *P* variances of the periodic intervals;
- β is the rate of false positive events, i.e., noise;
- $\alpha_{p}$ is the starting point of periodicity *p*;
- *M* is the generative model scheme.

The generative model scheme *M* is characterized by the priors for the distribution of the intervals, their mean values $\mu^{*}$, and their variances $\sigma^{*2}$. Following the generative approach in Equation (2), we assume that each event $s_{i}$ is generated according to one periodicity (except in the case of overlaps) or false positive noise β. Thus, the set *S* is the union of subsets $S^{p}$ of positive time stamps $s_{i}$ associated with periodicity $\mu_{p}$ or random noise β:

(3) $$S=S^{\mu_{1}^{*}}\cap S^{\mu_{2}^{*}}\cdots \cap S^{\mu_{P}^{*}}\cap S^{\beta }.$$

Without loss of generality, we parameterize the distribution of the intervals using the Gaussian distribution; any other distribution, such as those of the exponential family, would also be appropriate. In Section 2.1 and Section 2.2, we formulate two different model schemes: the *Clock Model* (M=C) and the *Random Walk Model* (M=RW).

### 2.1. Clock Model

The *“Clock Model”* describes periodic behavior governed by a fixed period $\mu_{p}^{*}$ with Gaussian noise, which does not rely on information from previous positive time stamps to compute the occurrence of the next event. For p=1,⋯,P and $i=1,\cdots ,N_{T}$, the events in $S^{p}$ are generated by:

(4) $$s_{i}^{p}=\alpha_{p}+\left(i\cdot \mu_{p}^{*}\right)+\epsilon ,\;\epsilon ∼N\left(0,\sigma_{p}^{*2}\right).$$

The number of events associated with uniformly distributed false positive noise is given as $\beta *|S^{\mu^{*}}|$ in the interval $\left[0,N_{T}\right]$.

In the Clock Model, the location of any event depends solely on its position in the time series and Gaussian noise around some regular interval but not on the previous time steps. Consequently, one can predict any future time step $s_{i+m}^{p}$ for m>0 with equal accuracy. This formulation generalizes the generative models found in much of the previous work on periodicity detection, such as in [15] and [12]. In their models, the objective is to identify a time slot si as a pair of a period (*l*) and an offset *i*, denoted by [l:i]. This approach is equivalent to finding a period $\mu_{p}^{*}$ and a starting point $\alpha_{p}$, with $\sigma^{*}=0$. However, this might be limiting in real applications, as it does not account for variability in event locations within the time series. To address this, we introduce Gaussian noise $\sigma^{*}$, with the case $\sigma^{*}=0$ being a special instance of the Clock Model.

However, the notion of a pacemaker, a component that imposes regular timing signals to synchronize events, is realistic only for certain systems, thereby motivating the development of the Random Walk Model.

### 2.2. Random Walk Model

The Random Walk Model exhibits the Markov property, meaning the temporal location of the next event depends on the current event’s temporal location and Gaussian noise. For p=1,⋯,P the events in $S^{p}$ are generated as follows:

(5) $$s_{i+1}^{p}=s_{i}^{p}+\mu_{p}^{*}+\epsilon ,\;\epsilon ∼N\left(0,{\left(i\sigma_{p}\right)}^{*2}\right).$$

The number of events associated with uniformly distributed false positive noise is given as $\beta *|S^{\mu^{*}}|$ in the interval $\left[0,N_{T}\right]$.

As the noise is Gaussian (and thus is identically distributed), the series of event time stamps in $S^{\mu_{p}}$, for p=1,⋯,P, describes a random walk. Therefore, the formulation in Equation (5) is referred to as the Random Walk Model (RWM).

The RWM has the characteristic that the variances $\sigma^{*}$’s accumulate with each subsequent time step. Consequently, the variance of the expected location of an event increases linearly with the distance from the current event. This assumption is essential and realistic for many real-life systems lacking a pacemaker, where predictability decreases with the number of steps. For example, given $s_{i}$, we can predict $s_{i+1}$ more accurately than $s_{i+10}$.

### 2.3. Inference

Given an event time series $X_{t}$, a straightforward approach to extract possible periodicities is to study the empirical histogram of all pairwise, forward-order inter-event intervals. For each event, we consider not only the interval to the next event (onset to onset) but also to all subsequent events.

The possible range of the intervals is defined by $\left(0,N_{T}\right)$. Please note, that in real applications, the actual range is smaller, as an interval needs to be observed a minimal number of times to be significant. We can estimate a histogram of expected forward-order inter-event intervals based on the generative models defined by Equations (4) and (5). This histogram is obtained by (i) analytically estimating the expected number of intervals for each $\mu_{p}^{*}\in \mu^{*}$, (ii) incorporating all intervals between any pair of events associated with different prime periodicities $\mu_{p}^{*}$ and $\mu_{q}^{*}$, and (iii) incorporating intervals due to noise (which can be performed analytically if the noise source is known; otherwise, an estimate is required). Comparing the empirical histogram with the parametric expectation defines the loss function used to identify the optimal underlying periodicities.

For every $\mu \in (0,N_{T})$, we define the function of interval counts D(μ) as:

(6) $$D\left(\mu \right)=\sum_{i,m>0}1_{s_{i+m}^{p}-s_{i}^{p}=\mu }.$$

Evaluating D(μ) for a given $X_{t}$ results in a histogram of all pairwise inter-event intervals.

The generative models provide a statistical model for the intervals. Therefore, we can estimate the expected number of intervals for $\mu \in (0,N_{T})$ in reference to a fixed periodicity $\mu_{p}^{*}$ and variance $\sigma_{p}^{*}$ as:

(7) $$\begin{array}{cc} E{\left[D\left(\mu \right)\right]}_{\mu_{p}^{*}} & =\sum_{i,m>0}E\left[1_{\mu }\right] \end{array}$$

(8) $$\begin{array}{cc} \; & =\sum_{i,m>0}P\left[s_{i+m}^{p}-s_{i}^{p}=\mu \right], \end{array}$$

where equality in Equation (7) is due to linearity of expectation and that in Equation (8) is due to the fact that for a random variable *A*, $E\left[1_{A}\right]=P\left[A\right]$. The distribution of all *m*-th order inter-event intervals depends on the specific generative model and can be written as

(9) $$P\left[s_{i+m}^{p}-s_{i}^{p}=\mu \right]=\frac{1}{\sqrt{2\pi \sigma_{p}^{*2}}}\exp[-\frac{{\left(\mu -m\mu_{p}^{*}\right)}^{2}}{2\sigma_{p}^{*2}}]$$

for the Clock Model, and as

(10) $$P\left[s_{i+m}^{p}-s_{i}^{p}=\mu \right]=\frac{1}{\sqrt{2\pi {\left(m\sigma_{p}^{*}\right)}^{2}}}\exp\left[-\frac{{\left(\mu -m\mu_{p}^{*}\right)}^{2}}{2{\left(m\sigma_{p}^{*}\right)}^{2}}\right]$$

for the Random Walk Model. For the latter, the variance grows linearly with the number of steps between events.

Further, we assume the starting point is zero, i.e., $\alpha_{p}=0$. For a time series of length $N_{T}$, Equation (8) can be rewritten in a more explicit form by expressing the indicator function as a definite quantity. Assuming no missing values in $\left(0,N_{T}\right)$, we should observe $\frac{N_{T}}{\mu_{p}^{*}}$ first-order intervals (m=1) in the time series, distributed according to the Gaussian probability density function (PDF) parametrized by $\sigma^{*}$ and $\mu_{p}^{*}$. For the second-order intervals (m=2), the scaling factor would be $\left(\frac{N_{T}}{\mu_{p}^{*}}-1\right)$, for m=3, $\left(\frac{N_{T}}{\mu_{p}^{*}}-2\right)$, and so on. Thus, for a single periodicity $\mu_{p}^{*}$, the expected value of D(μ) can be written for the Clock Model as:

(11) $$E{\left[D\left(\mu \right)\right]}_{\mu_{p}^{*}}=\sum_{m=1}^{\frac{N_{T}}{\mu_{p}^{*}}}\frac{const}{\sqrt{2\pi {\sigma_{p}^{*}}^{2}}}\exp\left[-\frac{{\left(\mu -m\mu_{p}^{*}\right)}^{2}}{2{\sigma_{p}^{*}}^{2}}\right]$$

and for the Random Walk Model as

(12) $$E{\left[D\left(\mu \right)\right]}_{\mu_{p}^{*}}=\sum_{m=1}^{\frac{N_{T}}{{\hat{\mu }}_{p}}}\frac{const}{\sqrt{2\pi {\left(m\sigma_{p}^{*}\right)}^{2}}}\exp[-\frac{{\left(\mu -m\mu_{p}^{*}\right)}^{2}}{2{\left(m\sigma_{p}^{*}\right)}^{2}}]$$

with $const=\frac{N_{T}}{\mu_{p}^{*}}-\left(m-1\right)$. Equations (12) and (11) therefore provide the expected values of the function D(μ) for counting all order intervals that might be observed for a single periodicity $\mu_{p}^{*}$ for the Random Walk and Clock Models, respectively.

In the case of multiple, overlapping periods, and/or false positive noise, the set of positive time stamps *S* consists of multiple sets: $S^{\mu_{1}^{*}}\cap S^{\mu_{2}^{*}}\cdots \cap S^{\mu_{p}^{*}}\cap S^{\beta }$. Since the affiliation of events to periodicities is unknown, we adapt our definition of D(μ) in Equation (6) by removing the superscript *p*:

(13) $$D\left(\mu \right)=\sum_{\forall i,m>0}1_{s_{i+m}-s_{i}=\mu }.$$

This modified operator D(μ) now counts the intervals not only between events from the same periodicity set but also between events in different sets and/or between noise events. We call the latter two “interaction intervals” and denote their contribution to D(μ) by:

(14) $$\zeta \left(\mu \right)=\sum_{\forall i,m>0}1_{s_{i+m}-s_{i}=\mu }.$$

This includes three possible scenarios (or their combination): (i) intervals between events from different periodicity sets, i.e., $s_{i}\in S^{\mu_{p}^{*}}$ and $s_{i+m}\in S^{\mu_{q}^{*}}$; (ii) intervals between events from any periodicity set and noise, i.e., $s_{i}\in S^{\mu_{p}^{*}}$ and $s_{i+m}\in S^{\beta }$; and (iii) intervals between noise events, i.e., $s_{i}\in S^{\beta }$ and $s_{i+m}\in S^{\beta }$.

The estimates in Equations (11) and (12) do not include these interaction intervals. Next, we discuss how to estimate $\zeta \left(\mu \right)$ and to account for the three cases explicitly. The distribution of the interaction intervals for all the three cases can be obtained in closed form by applying the convolution formula, which provides the distribution of the sum/difference of two interdependent discrete or continuous random variables [19]. For the Clock and the Random Walk Models, we obtain the following: For case (i), the interaction intervals between two periods, denoted as $\zeta_{pq}\left(\mu \right)$, are Gaussian distributed with a mean $\mu_{pq}=\mu_{p}-\mu_{q}$ and a variance $\sigma_{pq}^{2}=\sigma_{p}^{2}+\sigma_{q}^{2}$, where $\mu_{p}>\mu_{q}$ without loss of generality. For case (ii), the interaction intervals, denoted as $\zeta_{p\beta }\left(\mu \right)$, follow a Gaussian-like distribution, adjusted for the corresponding uniform support. For case (iii), the forward interaction intervals, denoted as $\zeta_{\beta }\left(\mu \right)$, follow the right sight of a triangle distribution. In this context, we can write $\zeta \left(\mu \right)$ as

(15) $$\zeta \left(\mu \right)=\zeta_{pq}\left(\mu \right)+\zeta_{p\beta }\left(\mu \right)+\zeta_{\beta }\left(\mu \right).$$

In real applications, the amount of noise in the data is unknown, and the exact formulation of (15) is not available, necessitating an approximation of $\zeta_{\beta }\left(\mu \right)$. We assume that the events contributing to the interaction intervals are uniformly distributed, an assumption that is not overly restrictive as demonstrated in Appendix A.1. Please note that we formulate the task of finding the optimal estimates for the periodicities by minimizing the distance (loss) between the observed interval differences D(μ) and the expected interval differences E[D(μ)] with respect to the chosen generative model. To obtain the expected differences, which include both interaction intervals and noise (whether for a Clock or Random Walk Model), we must explicitly approximate the interaction intervals caused by noise, as the amount of noise in real-world applications is unknown. In the next proposition, we provide an approximation, and hereafter, we derive a loss function that enables the estimation of multiple periodicities.

**Proposition** **1.** *For uniformly distributed events on $\left[1,N_{T}\right]$, the expected number of interaction intervals on the interval $\left[1,N_{T}\right]$ is given by:*

(16) $$E\left[\zeta \left(\mu \right)\right]=z\cdot \left(1-\frac{\mu }{N_{T}}\right)$$

*with a constant z, for every $\mu \in [1,N_{T}]$.*

**Proof.**  Consider two noise events $s_{i}^{\beta }$, $s_{j}^{\beta }$ each with a uniform probability mass function $P_{S}$ on the support $\left[1,\cdots ,N_{T}\right]$. The difference between the events $s_{j}^{\beta }-s_{i}^{\beta }=\mu \in \left[-N_{T},N_{T}\right]$ is a random variable whose probability mass function can be derived using the convolution formula for distributions [19]:

(17) $$P\left[s_{j}^{\beta }-s_{i}^{\beta }=\mu \right]=\sum_{s_{j}}P_{S}\left[s_{j}-\mu \right]P_{S}\left[s_{j}\right].$$

Given that $P_{S}$ is defined on $\left[1,\cdots ,N_{T}\right]$, the probability of $P_{S}\left[s_{j}-\mu <1\right]$ and $P_{S}\left[s_{j}-\mu >N_{T}\right]$ is zero. Therefore, we obtain:

(18) $$\sum_{s_{j}}P_{S}\left[s_{j}-\mu \right]P_{S}\left[s_{j}\right]=\left(N_{T}-\mu \right)\frac{1}{N_{T}}\frac{1}{N_{T}}$$

Finally, the probability mass function is a decaying function of the difference:

(19) $$P\left[s_{j}^{\beta }-s_{i}^{\beta }=\mu \right]=\frac{1}{N_{T}}\left(1-\frac{\mu }{N_{T}}\right).$$

If focusing on $|s_{j}^{\beta }-s_{i}^{\beta }|=\mu$, the right-hand side of Equation (19) must be multiplied by 2 due to symmetry. Next, we estimate the expectation of $\zeta \left(\mu \right)$:

(20) $$\begin{array}{cc} E[\zeta \left(\mu \right)]= & E[\sum_{\forall i,m>0}1_{s_{i+m}-s_{i}=\mu }] \end{array}$$

(21) $$\begin{array}{cc} = & \sum_{\forall i,m>0}E\left[1_{s_{i+m}-s_{i}=\mu }\right] \end{array}$$

(22) $$\begin{array}{cc} = & \sum_{\forall i,m>0}P\left[s_{j}^{\beta }-s_{i}^{\beta }=\mu \right]. \end{array}$$

The equality in Equation (21) is due to linearity of expectation, and that in Equation (22) is due to the fact that for a random variable *A*, the following equality holds: $E\left[1_{A}\right]=P\left[A\right]$. Inserting Equation (19) into Equation (22) results in:

(23) $$E\left[\zeta \left(\mu \right)\right]=\sum_{\forall i,m>0}\frac{1}{N_{T}}\left(1-\frac{\mu }{N_{T}}\right).$$

The number of all pairwise, forward-order differences for the noise events, with $n=N_{T}\beta =\left|S^{\beta }\right|$, is given as:

(24) $$\sum_{i}\left(n-i\right)=n^{2}-\frac{\left(n^{2}+n\right)}{2},$$

thus, we obtain:

(25) $$E\left[\zeta \left(\mu \right)\right]=\frac{2n^{2}-\left(n^{2}+n\right)}{2N_{T}}\left(1-\frac{\mu }{N_{T}}\right).$$

By setting $z=\frac{2n^{2}-\left(n^{2}+n\right)}{2N_{T}}$, we obtain Equation (16).    □

In real applications, the constant *z* cannot be estimated as the amount of false positives in the unknown a priori. An approximation $\hat{z}$ will be inferred from the data in Appendix A.1. For now, the expected number of interaction intervals is approximated via:

(26) $$E\left[\hat{\zeta }\left(\mu \right)\right]=\hat{z}\cdot \left(1-\frac{\mu }{N_{T}}\right).$$

In the case of multiple periodicities, due to linearity of expectation, the expected number of intervals over multiple periods is the sum of the expectations for D(μ) for each periodicity $\mu_{p}^{*}$ present in the data, plus the expected number of interaction intervals approximated by (14):

(27) $$E\left[D\left(\mu \right)\right]=\sum_{p=1}^{P}E{\left[D\left(\mu \right)\right]}_{\mu_{p}^{*}}+E\left[\zeta \left(\mu \right)\right].$$

Hereinafter, the first addend on the right-hand side is denoted as the deterministic parametric function $G_{M}\left(\mu ;\hat{\mu },\hat{\sigma }\right)$ for the Clock Model:

(28) $$G_{C}\left(\mu ;{\hat{\mu }}_{p},\hat{\sigma_{p}}\right)=\sum_{p=1}^{P}\sum_{m=1}^{\frac{N_{T}}{{\hat{\mu }}_{p}}}\frac{c_{p}}{\sqrt{2\pi {\hat{\sigma_{p}}}^{2}}}\exp\left[\frac{{\left(\mu -m{\hat{\mu }}_{p}\right)}^{2}}{2{\hat{\sigma_{p}}}^{2}}\right],$$

and the Random Walk Model:

(29) $$G_{RW}\left(\mu ;{\hat{\mu }}_{p},\hat{\sigma_{p}}\right)=\sum_{p=1}^{P}\sum_{m=1}^{\frac{N_{T}}{{\hat{\mu }}_{p}}}\frac{c_{p}}{\sqrt{2\pi {\left(m\hat{\sigma_{p}}\right)}^{2}}}\exp\left[\frac{{\left(\mu -m{\hat{\mu }}_{p}\right)}^{2}}{2{\left(m\hat{\sigma_{p}}\right)}^{2}}\right]$$

with $c_{p}=\left(\frac{N_{T}}{{\hat{\mu }}_{p}}-\left(m-1\right)\right)$.

Once we obtain estimates ${\hat{\mu }}_{p}$ and ${\hat{\sigma }}_{p}$ for the true periodicities $\mu_{p}^{*}$ and variances $\sigma_{p}^{*}$, and given a prior on the generating function (either Random Walk or Clock), we can write a loss function for our estimates as the difference between the empirical D(μ) and the parametric $G_{M}\left(\mu ;\hat{\mu },\hat{\sigma }\right)$. The loss function can be either the absolute error or a quadratic loss; since we have deterministic expectations, we focus on the absolute error as follows:

(30) $$\begin{array}{cc} L & =\sum_{\mu =1}^{N_{T}}\left|D\left(\mu \right)-E\left[D\left(\mu \right)\right]\right|, \end{array}$$

(31) $$\begin{array}{cc} \; & =\sum_{\mu =1}^{N_{T}}|D\left(\mu \right)-\left(\left[\sum_{p=1}^{P}E{\left[D\left(\mu \right)\right]}_{\mu_{p}^{*}}\right]+E\left[\zeta \left(\mu \right)\right]\right), \end{array}$$

(32) $$\begin{array}{cc} \; & \approx \sum_{\mu =1}^{N_{T}}\left|D\left(\mu \right)-G_{M}\left(\mu ;\hat{\mu },\hat{\sigma }\right)-E\left[\hat{\zeta }\left(\mu \right)\right]\right|. \end{array}$$

Finally, for either the Clock Model or the Random Walk Model, the aim is to find a set of periodicities and variances that minimize the corresponding loss. A straightforward approach would consider all possible combinations of acceptable periodicities and variances, where the optimal combination minimizes the loss.

However, such an approach is computationally infeasible. Therefore, the following section outlines the Gaussian Mixture Periodicity Detection Algorithm (GMPDA).

## 3. GMPDA

Given an event time series $X_{t}\in R^{\left(1\times N_{T}\right)}$ the aims are (i) to extract an estimate $\hat{\mu }$ of the true generating periodicities $\mu^{*}$, (ii) to infer $\sigma^{*}$, and (iii) to test the fit of the chosen generative model *M*. The GMPDA provides a method to learn the parameters of the generative function of $X_{t}$ accurately and efficiently by minimizing the loss L defined in Equation (32). The GMPDA is open-source and available on https://github.com/nnaisense/gmpda (accessed on 18 July 2024). The GMPDA is based on comparing D(μ), the empirical distribution of the intervals observed in the time series $X_{t}$, with parametrized estimates of its generative function $G_{M}\left(\hat{\mu },\hat{\sigma }\right)$, plus the contribution coming from the interaction intervals, using the loss function (32). The main steps of the GMPDA for estimating the optimal parameters $\hat{\mu },\hat{\sigma }$ are outlined in Algorithm 1.
**Algorithm 1:** Main Steps of the GMPDA._1_Extract event time stamps: *S* ← where $X_{t}=1$_2_Compute intervals D(μ) from *S*, with respect to Equation (A2) and subtract ζ(μ), estimated with respect to Equation (A1)_3_Identify candidates periods using integral convolution_4_Initialize and optimize variance for candidates periods_5_Find optimal combination of periodicities, which minimize the loss defined in (32)_6_Update loss and sigma with respect to optimal periodicities

After extracting the event time stamps, the GMPDA computes D(μ) with respect to Equation (A2) and subtracts the approximated contribution from the interaction events (step 2 of Algorithm 1). The approximation of the length of interaction intervals is either limited by the minimal expected periodicity or by a user-defined parameter, denoted as noise_range. The estimation of the approximation is outlined in Appendix A.1. For the estimation of D(μ) and the loss, the range for μ is limited by the parameter loss_length, mainly due to the flattening of the Gaussian distribution with increasing variance for the Random Walk Model. A detailed discussion can be found in Appendix A.2.

In the *third step*, the GMPDA estimates a set of candidate periodicities using a heuristic approach since computing $G_{M}\left({\hat{\mu }}_{p},\hat{\sigma }\right)$ for all possible ${\hat{\mu }}_{p}$ is computationally expensive. The heuristic approach iteratively searches for periodicities $\hat{\mu }$ by performing ”integral convolutions” on D(μ) in each iteration. The convolution smooths the function for extracting periods that explain the time series. The maximum number of candidates is controlled by parameter max_candidates, and the maximum number of iterations by the parameter max_iterations. This heuristic approach is described in detail in Appendix A.2.

In the *fourth step*, the GMPDA performs least-squares curve fitting to refine the initial guess for $\hat{\sigma }$. This step is optional and can be controlled by the parameter curve_fit. The curve fitting procedure is described in Appendix A.3.

In the *fifth step*, the GMPDA computes the function $G_{M}\left({\hat{\mu }}_{p},\hat{\sigma }\right)$ for all combinations of candidate periodicities and corresponding variances. It then selects the set of ”prime periodicities” ${\hat{\mu }}^{*}$ that minimizes the loss, defined and explained in Appendix A.4. In the final, sixth step, the loss and $\hat{\sigma }$ are updated with respect to optimal periodicities.

## 4. Performance Evaluation on Test Cases

This section evaluates the capacity of the GMPDA to detect periodicities $\mu^{*}$ and variances $\sigma^{*}$ on synthetic time series generated according to Clock and Random Walk Models. The performance of the GMPDA in detecting periodicities $\mu^{*}$ was compared with other periodicity detection algorithms, including FFT, autocorrelation with FFT, histogram with FFT, and E-periodicity (the alternative algorithms were implemented in MATLAB). For all algorithms, the minimal and maximal considered period lengths were set to 10 and 350, respectively. The corresponding code is available on https://github.com/nnaisense/gmpda (accessed on 18 July 2024)). These specific algorithms are described below.

**GMPDA**: We used the baseline algorithm described in Algorithm 1 with $\hat{\sigma }$ set equal to $\sigma^{*}$ (i.e., $\sigma^{*}=\log\left(\mu \right)$) and no non-linear curve fitting.

GMPDA $\sigma^{*}$ unknown: The algorithm is initialized with $\hat{\sigma }=int\left(\log\left(10\right)\right)$, which is the minimal possible value for $\mu^{*}$. For the above GMPDA configurations, the algorithm searches for maximal $|\mu^{*}|+2$ periodicities. Please note that we have chosen here to use a different sigma for the application of the GMPDA with curve fitting (i.e., $\hat{\sigma }=\sigma^{*}$) compared to the GMPDA without curve fitting ($\hat{\sigma }=int\left(\log\left(10\right)\right)$). In real applications, sigma is unknown and would be supplied as the best guess available. The GMPDA with curve fitting tries to optimize the initial estimate of sigma once the candidate periodicities are identified. If no non-linear curve fitting is deployed, we suggest running the algorithm multiple times for a range of possible $\hat{\sigma }$ values, and the optimal $\hat{\sigma }$ can then be chosen with respect to the lowest loss.

**FFT**: This is a Power Spectral Density Estimates approach [1]. In the case of a single periodicity, the frequency with the highest spectral power is selected as the prime periodicity. In the case of multiple periodicities, the frequencies $|\mu^{*}|$ with the highest spectral power are selected as the true periodicities.

**Autocorrelation with FFT**: The Autocorrelation Function (ACF) estimates how similar a sequence is to its previous sequence for different lags and then uses the lag that maximizes ACF as the predicted period [5]. Since all integer multiples of true periods will have the same function value, an FFT is applied to the ACF to select the frequencies with the highest spectral power as the true periodicities. In the case of multiple periodicities, the frequencies with the highest spectral power are selected as the true periodicities.

**Histogram with FFT**: This is an FFT applied to the histogram of all forward differences in the time series D(μ) [20]. In the case of multiple periodicities, the frequencies with the highest spectral power are selected as the true periodicities.

**E-Periodicity**: We implement the method presented in [12], which computes a “discrepancy score” for each possible periodicity, i.e., the number of intervals between events that are equal to the candidate periodicity. To detect multiple periods, we select the top $|\mu^{*}|$ candidate periods from the discrepancy function.

There are several conceptual differences and similarities between the GMPDA and the alternative algorithms: the GMPDA, like all the methods listed above, computes frequencies/periodicities based on the observed intervals between positive observations in the time series. For data that follow a Clock Model, variance in intervals can be handled using regression frameworks for ACF and spectral methods for FFT. However, for very small or large variations in intervals, parametrized by $\sigma^{*}$ in the Random Walk Model, these methods may struggle—particularly for multiple periodicities—due to the linear increase in variance. E-periodicity and histogram methods are likely to show decreased performance for variable intervals, as they lack specific mechanisms to handle interval variance, which is particularly problematic for time series following the Random Walk Model.

The GMPDA is designed for multiple periodicity detection, and its loss function explicitly targets finding all periodicities present in the data. Once the set of candidate periodicities is identified, the GMPDA checks all possible combinations of periodicities and selects the one with the smallest loss.

ACF and FFT are accepted methods for hierarchical frequency detection, but they lack a “stopping criteria” to determine the number of significant periodicities in the time series. This can lead to the over- or underestimation of the number of periodicities. In our test cases, we always selected the top |μ∗| frequencies as the true periodicities, likely overestimating the accuracy of these methods since there is no way to know this number without prior knowledge of the generative mechanism. Additionally, the E-periodicity and histogram methods are not explicitly designed for multiple periodicity detection and lack mechanisms for handling noise in intervals.

Conceptually, the GMPDA differs from classical Gaussian Mixture and Hidden Markov (HMM) approaches. All three methods aim to fit the shape of a distribution described by the corresponding histogram of the data. However, the GMPDA generative models account for peaks in the histogram at prime periods and their integer multiples, combining these peaks for a better estimate. Classical MM/HMM models do not use this information; instead, they try to fit all peaks individually if the number of mixture models *K* is large, or average them if *K* is small, leading to biased results.

In the following, we compare the performance of the above-described algorithms on a large set of generated test cases.

### 4.1. Test Cases

The performance of the GMPDA was evaluated on a wide range of test cases for the Clock Model and the Random Walk Model. The test cases systematically varied the following model parameters: periodicity μ, variance σ, noise β, and the number of events *n*. These generative model parameters influence the histogram of inter-event intervals, which is the input data for all applied algorithms.

**Figure 1:**
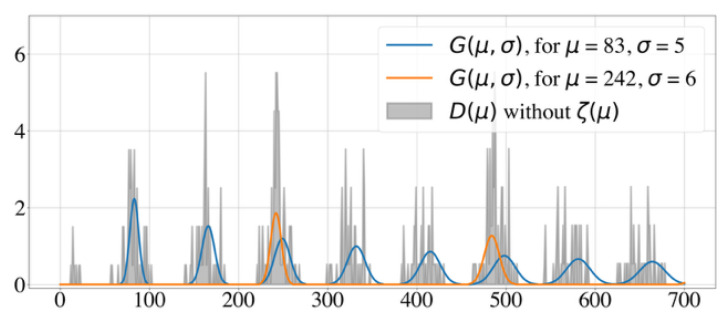
Example of a well-posed test case with two underlying periodicities and no noise. Histogram of the intervals $D\left(\mu \right)-\hat{z}$ and generative curves G(μ,σ) for the Random Walk Model with n=30 and β=0.

**Figure 2:**
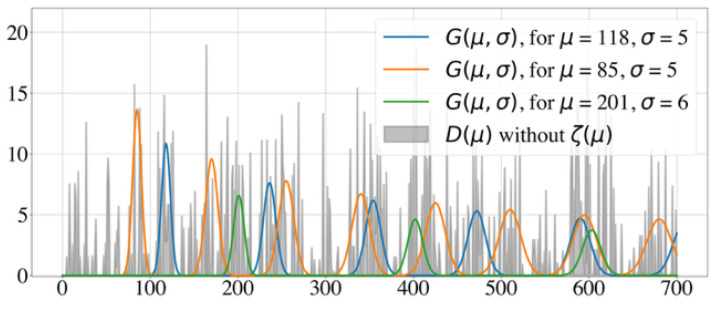
Example of an ill-posed test case with a signal-to-noise ratio of 1:2. Histogram of the intervals $D\left(\mu \right)-\hat{z}$ and generative curves G(μ,σ) for the Random Walk Model with n=100 and β=2.

To better understand how these model parameters influence the histogram, we show two illustrative test cases with different model parameters. Figure 1 shows a well-posed test case, with two underlying periodicities and no noise, while Figure 2 displays an ill-posed test case, where the signal-to-noise ratio is 1:2. Identifying underlying periodicities in the latter case requires advanced analysis of the histogram.

The following analyses examined an extensive range of test cases to study the limitations of the presented GMPDA and the alternatives described in Section 4.

#### Configurations

For the configuration of the test cases, we considered the following values for the model parameters:

- $\sigma^{*}\in \left\{1,\log\left(\mu \right),\frac{\mu }{p}\right\}$, with p=16,8,4,3,
- n∈{10,30,50,100,300,500},
- β∈{0,0.1,0.5,0.7,1,2,4,8}.

Please note that test cases with σ=log(μ) represent scenarios where no σ optimization is required, as σ=log(μ) is the default initialization in the GMPDA. Small values for *n* and large values for σ,β were chosen to investigate the limits of the periodicity detection algorithms.

For every combination of $\sigma^{*}$, β, and *n*, we generated 100 event time series with randomly drawn $\mu^{*}\in \left[10,350\right]$. For test cases with multiple periodicities, we enforced the difference between the involved periodicities to be bigger then log(μ). Otherwise, the generative curves become indistinguishable too quickly, making multiple periodicity detection too ill-posed.

The combination of the above model parameter settings resulted in 28,800 test cases for each generative model. All algorithms were applied to identify the underlying periodicities for every generated test case.

An identified periodicity is considered correct if it lays within $\mu^{*}\pm 0.5\cdot \sigma$, where $\mu^{*}$ is the true periodicity and σ the corresponding variance. For instance, for the cases $\mu^{*}=15,\;\sigma =2$ and $\mu^{*}=350,\;\sigma =44$, a guess of μ within 15±1 and 350±22, respectively, would be considered an accurate detection.

Thus, for a fixed configuration of the parameters σ, β, and *n*, the performance of the algorithms is measured by accuracy, which is the average number of correctly identified periodicities (across the 100 generated test cases) with a value between zero and one.

In the following, we first present the results for $|\mu^{*}|=1$ and identify valid ranges for *n*, β, and σ. Second, within the valid range, we compare the performance of the GMPDA to that of the other algorithms for $|\mu^{*}|=1,2,3$.

### 4.2. Performance with Respect to $|\mu^{*}|=1$

#### 4.2.1. GMPDA Performance

In this section, we focus on the performance of the GMPDA with respect to $|\mu^{*}|=1$ to determine realistic limits for σ,β, and the number of events.

**Figure 3:**
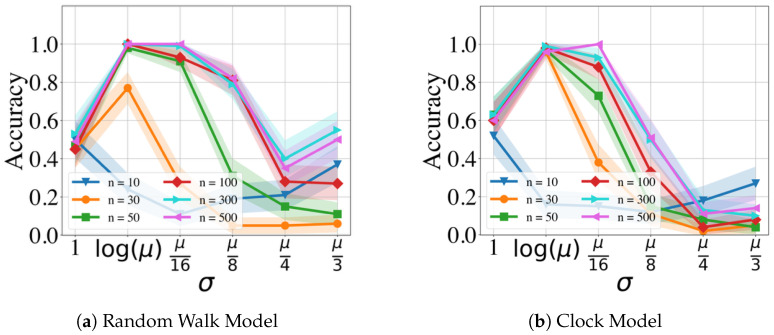
Performance of the GMPDA *without* curve fitting for the Random Walk Model (**a**) and for the Clock Model (**b**), with β=0 and |μ|=1 and varying number of events (n).

**Figure 4:**
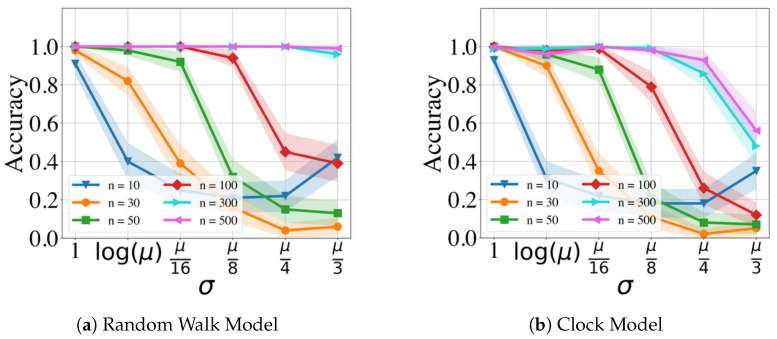
Performance of the GMPDA *with* curve fitting for the Random Walk Model (**a**) and for the Clock Model (**b**), with β=0 and |μ|=1 and varying number of events (n).

Figure 3 and Figure 4 display the performance of the GMPDA for fixed β=0 and |μ|=1 with varying values of σ and different numbers of events *n*, without and with curve fitting, respectively. Curve fitting is an optional step in the GMPDA that helps optimize the estimate for σ once the algorithm has identified the candidate periodicities. The confidence intervals (CI) in all the following figures (if present) are estimated as $\bar{x}\pm 1.96$ SEM, where $\bar{x}$ is the mean and SEM is the standard error of the mean.

The results in Figure 3 and Figure 4 show, as expected, that accuracy is decreased with increasing σ and decreasing the number of events. In other words, with increasing variance, more events are required for an accurate detection.

The figures also compare the performance of the GMPDA with and without curve fitting. The GMPDA without curve fitting performed worse, except in the case of σ=log(μ). This behavior can be explained as follows: in the algorithm, the default initialization value of σ is log(μ), and therefore for this configuration, the GMPDA without curve fitting worked with a known sigma. In all the other cases, the GMPDA with curve fitting provided better results.

**Figure 5:**
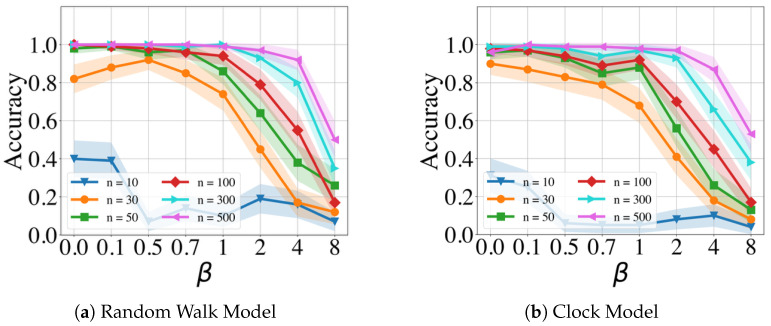
Performance of the GMPDA with curve fitting for the Random Walk Model (**a**), and for the Clock Model (**b**), with |μ|=1 and σ=log(μ) across varying levels of uniform noise beta and number of events.

Next, to compare the effect of noise, we restricted our evaluation to the GMPDA with curve fitting due to its better performance. Please note that the comparison between results with and without curve fitting can be found in Appendix B.1. Further, we focused on the case of $|\mu^{*}|=1$ and known σ, which can be viewed as an *ideal* scenario, as only μ needs to be estimated. For this ideal case, we compared the effect of varying noise levels across a varying number of events on detection accuracy. Figure 5 shows the performance of the different algorithms with respect to increasing the amounts of noise in the time series, for the case with |μ|=1 and σ=log(μ), and separately for Random Walk and the Clock Models.

For the Random Walk Model (Figure 5a), performance was acceptable for signals with n≥300 and noise up to β=4; for n≤300, performance dropped below 0.75 already for β≥2. In comparison, the Clock Model was substantially more sensitive to noise (Figure 5b) with acceptable results only for β≤1.

In summary, in cases where the actual variance is unknown, GMPDA with curve fitting outperformed the GMPDA without curve fitting. The GMPDA was not suited for cases with fewer than 50 events. The GMPDA performance increased with the number of events. The GMPDA could also handle moderate to high amounts of noise, and we show in the next section how this compares to other periodicity detection algorithms.

#### 4.2.2. Comparison with Alternative Periodicity Detection Algorithms

Next, we compared the GMPDA (with curve fitting) algorithm to other periodicity detection algorithms regarding their performance under varying conditions of noise and variance. As noise and variance increase, the histograms of the inter-event intervals analyzed by all algorithms become less informative, making the peaks that indicate periodicities less identifiable. Therefore, we investigated the sensitivity to noise and different variances used for generating the periodicities. We first examined the effect of varying levels of variance σ for cases where no noise was present, i.e., β=0.

**Figure 6:**
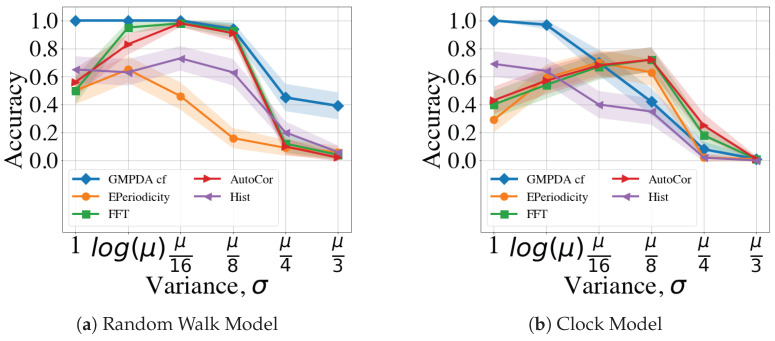
Comparison of the GMPDA to alternative algorithms: for the Random Walk Model (**a**), and for the Clock Model (**b**). Accuracy is plotted for different levels of variance σ for cases with one period (|μ|=1), no noise (β=0) and number of events, n=100.

The results for all algorithms and n=100 are shown in Figure 6. The results for different numbers of *n*, averaged over all levels of β, can be found in Appendix B.3. For the Random Walk Model, the GMPDA was very accurate up to $\sigma =\frac{\mu }{8}$. Interestingly, all other algorithms performed worse when variance was very small ((σ=1 and σ=log(μ)), a case where the GMPDA excelled. FFT and AutoCor converged to the accuracy bound given by the GMPDA for σ>1, while the accuracy of E-periodicity and Hist had its maximum of about 0.8. For all methods, the performance dropped for $\sigma \geq \frac{\mu }{8}$. This behavior is distinctive for the Random Walk Model, where the variance increases with every step, causing the generative distributions to start overlapping more quickly with larger variance. Performance was generally lower for the Clock Model, which was also more sensitive to increases in the variance. The GMPDA was sufficiently accurate only for σ=1 and σ=log(μ), with a distinct drop in performance with increased variance. For the other algorithms, except the histogram method, performance initially increased with increasing variance up to $\sigma =\frac{\mu }{8}$ and then declined sharply.

**Figure 7:**
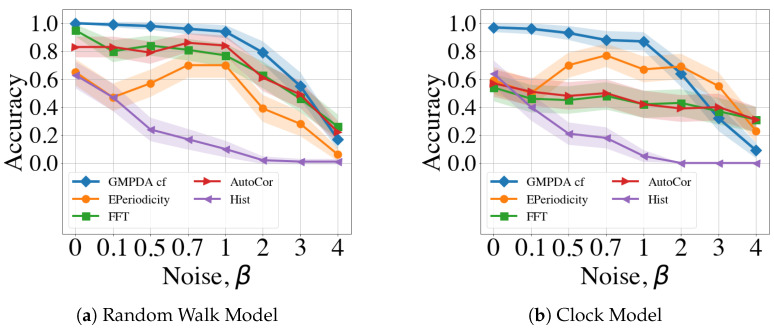
Comparison of the GMPDA to alternative methods: for the Random Walk Model (**a**), and for the Clock Model (**b**). Accuracy is plotted against increasing levels of noise β for cases with one period (|μ|=1), known variance, i.e., σ=log(μ)) and number of events, n=100.

Next, we evaluated the performance of all methods with respect to increasing levels noise, with the results shown in Figure 7. For these analyses, the variance was fixed to σ=log(μ)) and number of events to n=100. The plots for all numbers of *n* can be found in Appendix B.2. For the Random Walk Model, the GMPDA was insensitive to noise up to β=1, with performance decreasing linearly thereafter. The performance of FFT and AutoCor mirrored that of the GMPDA with slightly lower levels of accuracy. Notably, E-periodicity’s performance increased up to β=1 and then declined, while Hist was very sensitive to all levels of noise and performed worse than all other algorithms.

For the Clock Model, the GMPDA behaved similarly, while the performance of the other methods was more sensitive to noise, and accuracy was generally lower than for the Random Walk Model.

The presence of moderate noise (i.e., with β∈[0.1,0.7]) did not affect performance, except for E-periodicity, where performance increased for noise levels up to β=2.

The maximal noise levels that the algorithms could handle were not higher than two β≤2, i.e., a signal-to-noise ratio of 1:2, one periodic event to two noise events.

**Figure 8:**
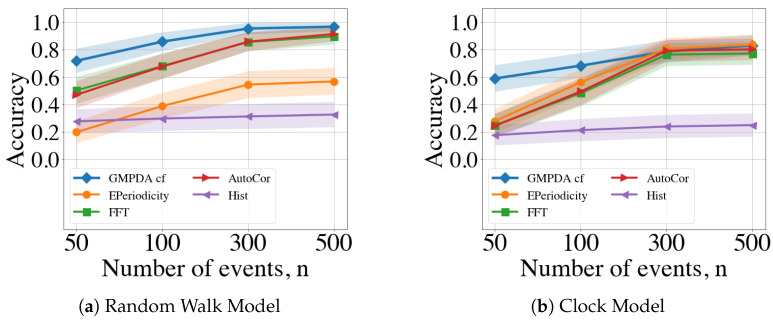
Comparison of the GMPDA to alternative methods for the Random Walk Model (**a**), and for the Clock Model (**b**). Accuracy is plotted against the number of events averaged over $\sigma =\{1,log\left(\mu \right),\frac{\mu }{16},\frac{\mu }{8}\}$ and β≤2.

In conclusion, we averaged performance over all acceptable values of noise and variance (i.e., $\sigma =\{1,log\left(\mu \right),\frac{\mu }{16},\frac{\mu }{8}\}$ and β≤2). The results are shown in Figure 8. Overall, the detection of a single periodicity was increasingly accurate with an increasing number of events for all methods and both the Random Walk and Clock Models (see Figure 8). For both models, the periodicity detection with the Hist algorithm had very low accuracy with a maximal performance of less than 0.4.

For the Random Walk Model, the GMPDA outperformed alternative approaches, with accuracy converging to one as the number of events increased, and even for n = 30, its performance was larger than 0.75. FFT/Autocor achieved similar performance when the number of events was larger than 300. In contrast, EPeriodicty’s performance for the Random Walk Model was relatively poor, with a maximum of 0.6 for 500 events.

For the Clock Model, the GMPDA outperformed alternatives when the number of events was smaller than 300. For more than 300 events, the performance of all approaches, except Hist, became equally good.

### 4.3. Performance with Respect to $|\mu^{*}|>1$

This section compares the performance of the GMPDA (with and without curve fitting) to that of the alternative methods for multiple periodicity detection, focusing on the set of sensible simulation parameters identified in Section 4.2.2. These parameters are n=50,100,300,500, $\sigma =\{1,log\left(\mu \right),\frac{\mu }{16},\frac{\mu }{8}\}$, and β≤1, resulting in 8000 test cases for each setting of |μ|=2 and |μ|=3 for every generative model. For comparison, the performance is summarized over n,μ,σ, and β, visualized here as a histogram, where the *x*-axis displays the number of correctly detected periodicities and the *y*-axis the number of test cases.

**Figure 9:**
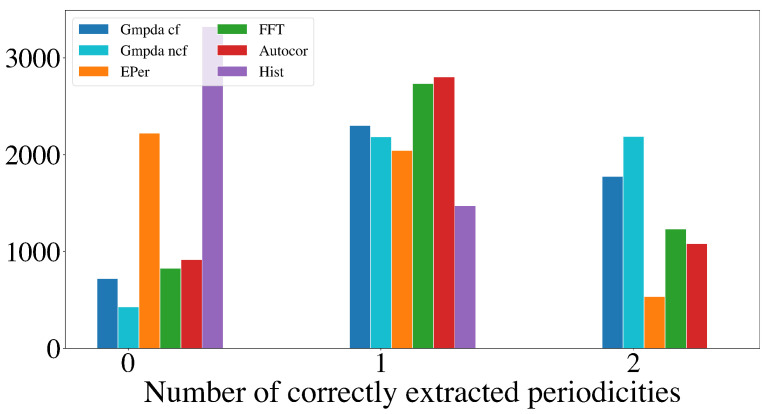
Detection of multiple periodicities (|μ|=2) quantified as the number of correctly extracted periodicities by the GMPDA and alternative methods for the Random Walk Model.

**Figure 10:**
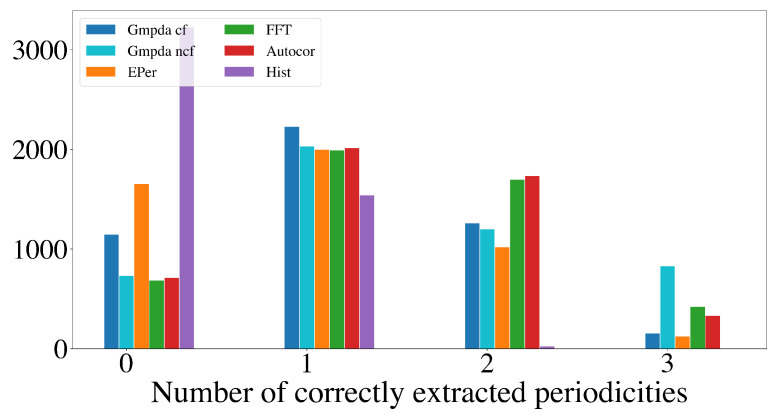
Detection of multiple periodicities (|μ|=3) quantified as the number of correctly extracted periodicities by the GMPDA and alternative methods for the Random Walk Model.

**Figure 11:**
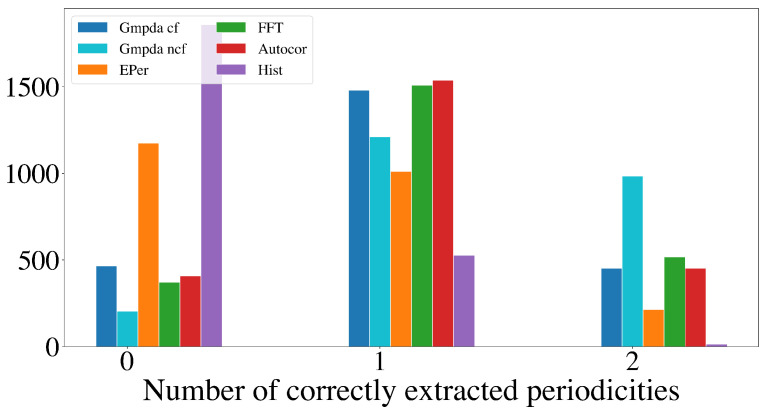
Detection of multiple periodicities (|μ|=2) quantified as the number of correctly extracted periodicities by the GMPDA and alternative methods for the Clock Model.

**Figure 12:**
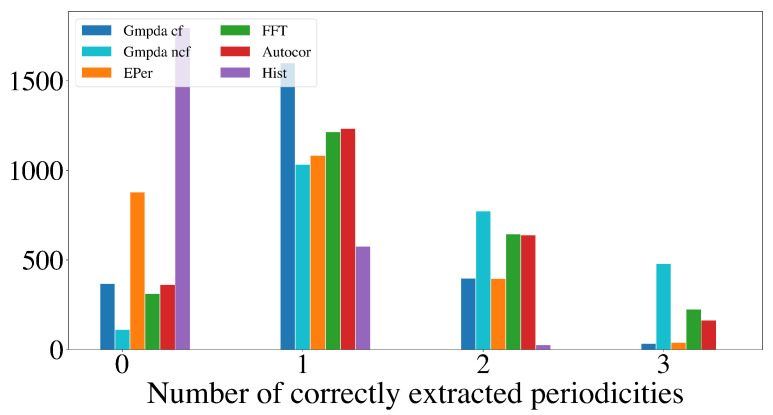
Detection of multiple periodicities (|μ|=3) quantified as the number of correctly extracted periodicities by the GMPDA and alternative methods for the Clock Model.

Figure 9 and Figure 10 show the results for the Random Walk Model, and Figure 11 and Figure 12 show the results for the Clock Model for |μ|=2 and |μ|=3, respectively.

For the case with two periodicities, |μ|=2, the GMPDA outperformed the alternative methods, both with and without curve fitting. Interestingly, the GMPDA without curve fitting performed slightly better, suggesting that the current sigma optimization might require further development.

The detection of three periodicities, |μ|=3, was challenging for all methods as shown in Figure 10 and Figure 12. One possible explanation is that with more periodicities, there are more interaction intervals, i.e., intervals between the periodic events from different periodicities. Furthermore, at least for the Random Walk Model, the histogram becomes less identifiable as σ grows with each subsequent step, flattening out the distribution responsible for the events. This effect is amplified when more than one periodicity is present. We conclude that the GMPDA in the current version is not well suited for detecting more than two periodicities.

### 4.4. Computational Performance

The computational performance (CPU time) of the GMPDA was evaluated across different experiments. For this purpose, time series were generated for every combination of the following model parameters: $|\mu^{*}|=\left[1,2,3\right]$, events per periodicity = [50,100,300,500], $\sigma^{*}=\left[\log\left(\mu \right)\right]$, β=[1]. The GMPDA was executed for each time series, and the computational/execution time was determined using the Python module timeit with 100 executions. For the generated test cases, we tested them with the following GMPDA configurations (described in Section 3): loss_length=[400,800,1200] and max_periods=[|μ|+2]. The remaining parameters were fixed at $L_{min}=5$, max_iterations=5, max_candidates=15, noise_range=5, loss_tol_change=0.01.

**Figure 13:**
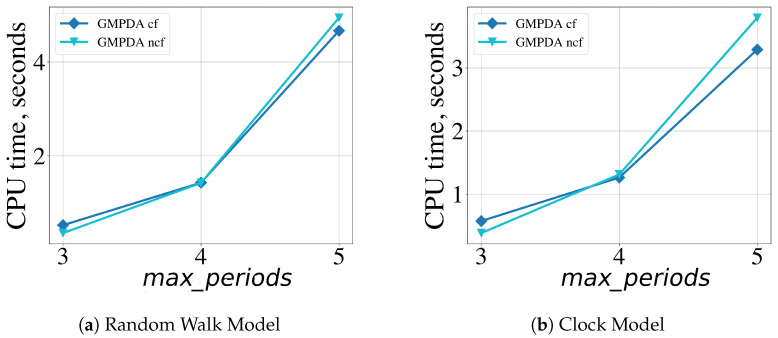
Effect of the GMPDA parameter max_periods, the maximum number of searched for periodicities, on computational performance averaged over 1200 executions for the GMPDA, with (dark blue symbols) and without curve fitting (light blue symbols).

Our analysis shows that the computational performance strongly depended on the maximum number of allowed periodicities, max_periods. The CPU time for both models (averaged over the number of executions, number of events n, and loss length) is shown in Figure 13. All other parameters had a comparatively minor influence on the performance. In additional experiments not shown here, we also investigated the influence of noise β on the computational performance of the algorithm.

The results indicated that although, on average, the CPU time increased slightly with increasing noise β, the influence was minimal when compared to the maximum number of allowed periodicities, max_periods. Finally, the maximal number of candidates periods max_candidates also affected the CPU time: a lower max_candidates resulted in faster execution time but decreased the accuracy of the algorithm.

The proposed GMPDA is optimized by vectorizing all major computations. Due to the hierarchical structure of the algorithm, the computational time will depend on the maximum number of periodicities. The computationally costly part arises from the curve fitting optimization, which is negligible as shown in Figure 13. The algorithm’s memory requirements are linearly dependent on the length of the considered time series. Therefore, the algorithm is scalable and applicable for real data applications.

### 4.5. Summary

We have evaluated the performance of the GMPDA across a large set of test cases, covering different configurations of the Random Walk and the Clock Models. Our main findings are as follows: First, for time series following the Random Walk Model, the GMPDA outperformed alternative algorithms. Second, for time series following the Clock Model, the GMPDA outperformed alternative methods in cases with low variance of the inter-event intervals. Third, all algorithms struggled to identify more than two periodicities.

Additionally, we analyzed the sensitivity to critical simulation parameters across the different algorithms and found that both sigma and the number events emerged as the strongest determinants of periodicity detection accuracy. The details of the sensitivity analysis can be found in Appendix B.4.

## 5. Real Application

Finally, we applied the GMPDA to real data, specifically to the recording of leg movements during sleep from the publicly available MrOS data set [21,22,23,24,25].

From 2905 available sleep recordings in community-dwelling men 67 years or older (median age 76 years), we considered all recordings with at least 4 h of sleep, a minimum of 10 leg movements and 10 arousals, and adequate signal quality based on various parameters in the MrOS database. This resulted in 2650 recordings satisfying our inclusion criteria, from which we randomly selected 100 recordings for this real application case.

We chose to examine leg movements during sleep because it is known that in a relatively large proportion of the population (up to 23% [26]), these leg movements tend to occur in a periodic pattern, known as periodic leg movements during sleep (PLMS) [27], with a typical inter-movement interval around 20 to 40 s [28]. We, therefore, expected to find some amount of periodicity in this data set, making this analysis a real-life positive control.

We applied the GMPDA to both raw and preprocessed data. In the preprocessing step, the time series of leg movements for each subject was segmented into *sleeping* bouts according to the following criteria: Each bout (i) contained only sleep interrupted by not more than 2 min of wakefulness, (ii) lasted at least 5 min, and (iii) contained at least four leg movements. This resulted in 579 sleep bouts from the 100 recordings where the GMPDA was applied independently to each bout. The number of events was less than 100 for 85% of the bouts, and for those, the average bout length was 2572 s.

### 5.1. GMPDA Configurations

The following GMPDA parameters were fixed for both data sets (i.e., whole night data and sleep bout data): $L_{min}=5$, $L_{max}=200$, max_iterations=5, max_candidates=15, loss_length=400, max_periods=5, noise_range=5, loss_tol_change=0.1. We chose a tolerance value for a decrease in the loss of 0.1, meaning additional periodicities are only considered if their inclusion results in a change in loss greater than this tolerance value. This value is substantially higher than in the simulated examples (0:01) because, in this first real-life application, we aimed to generate robust results given the expected noise in the data. In this context, the results presented here and the periodicities identified can be seen as ”low-hanging fruit”. Moreover, the detection of additional periodicities would be expected with different GMPDA parameters.

**Figure A7:**
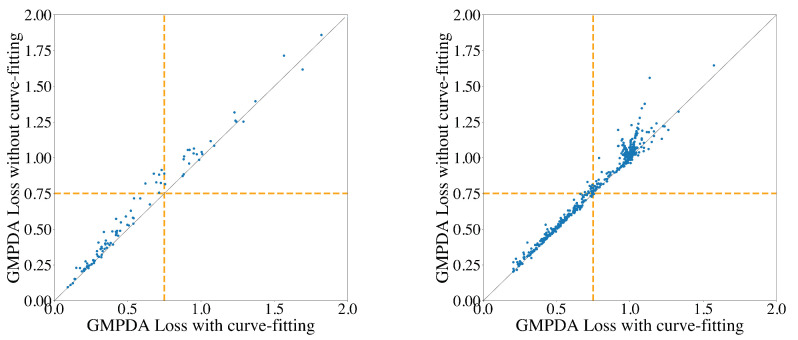
Comparison of GMPA loss with and without curve fitting for individual nights (**left panel**) and single sleep bouts (**right panel**).

For the MrOS data set, we assumed a Random Walk Model, which we applied both with and without the curve fitting of the variance parameter $\hat{\sigma }$. Consistent across all single records, the curve fitting approach identified periodicities with a lower loss, so we will describe only the curve fitting results in the following. The GMPDA loss with and without curve fitting is compared in Appendix B.5, Figure A7.

### 5.2. Reference Loss

The GMPDA identifies the periodicity with minimal loss. However, even if minimal, this loss might still be numerically significant. In a real-life application where it can be assumed that some of the time series do not contain periodic events, it is necessary to identify loss values that do not support the existence of periodicities in the data. We address this issue: we constructed a reference loss, derived from the minimal GMPDA loss returned for times series that contain only random noise.

For the MrOS data set, the length of the included bouts and the number of events ranged from 300 to 24,000 seconds and 5 to 430 events, respectively. To obtain an overall reference loss, we constructed 100 noisy bouts with uniformly distributed events for all different combinations of the number of events [10; 30; 50; 100; 200; 400] and length of the bout [500; 1000; 2000; 4000; 8000; 16,000]. Applying the GMPDA to each combination, we obtained an empirical distribution of loss values for cases where the events were generated randomly and did not exhibit any clear periodic pattern. The global MrOS reference loss is set to the 0.01 quantile of this distribution, corresponding to a value of 0.74468, rounded to 0.75 in the following.

Additionally, we estimated a local reference loss for each bout in the MrOS data set by generating 100 time series with the bout-specific length and the number of events and taking 0.01 quantile of the resulting loss distribution. A significant periodicity was identified when the GMPDA loss for this bout was lower than the local reference loss. However, the significant periodicities obtained with local and global reference losses did not differ significantly, and for simplicity, we focus on the results obtained for a global reference loss of 0.75.

### 5.3. Results

**Figure 14:**
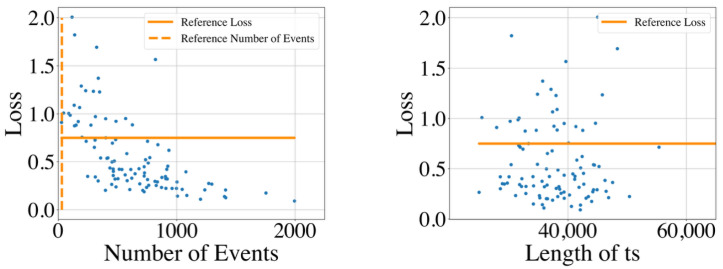
The GMPDA loss for 100 whole night time series plotted against the number of events (**left panel**) and length of time series (ts, in seconds, **right panel**).

**Figure 15:**
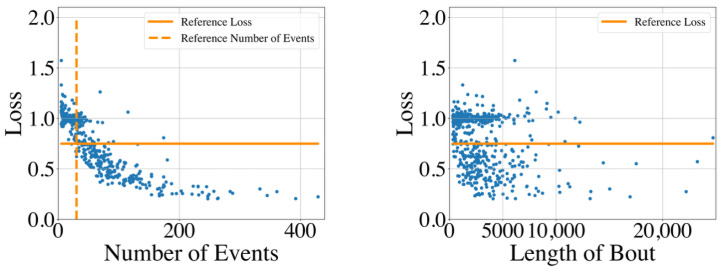
The GMPDA loss for 579 sleep bouts of at least 5 min plotted against the number of events (**left panel**) and the length of the sleep bout (in seconds, **right panel**).

The distribution of the GMPDA model loss for all time series is shown in Figure 14 for the whole night recording and in Figure 15 for the single sleep bouts. The figures suggest that the GMPDA loss did not systematically change with the length of the times series. However, the loss tended to decrease with the number of events in the time series. More specifically, as already seen in the simulation experiments, for time series with a low number of events, the resulting loss was not distinguishable from the loss found for non-periodic time series. The left panel of Figure 15, which shows the distribution of the loss for the number of events in the MrOS data set, could also suggest a minimum number of events needed for the GMPDA to detect a significant periodicity in this data set. For the records selected here, no significant periodicity was detected for any bout with fewer than 30 events (see reference number of events in Figure 15). Further analysis with other records from the same data set and new data sets is needed to determine whether this reference number constitutes an absolute threshold for biomedical event data.

**Figure 16:**
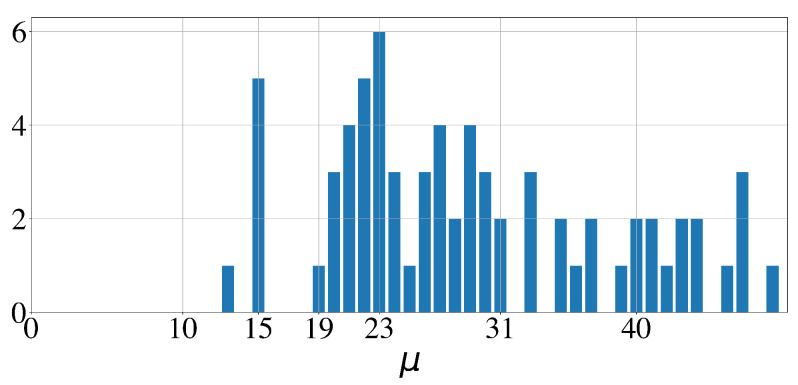
Histogram of significant periodicities identified in 100 whole night time series by the GMPDA.

**Figure 17:**
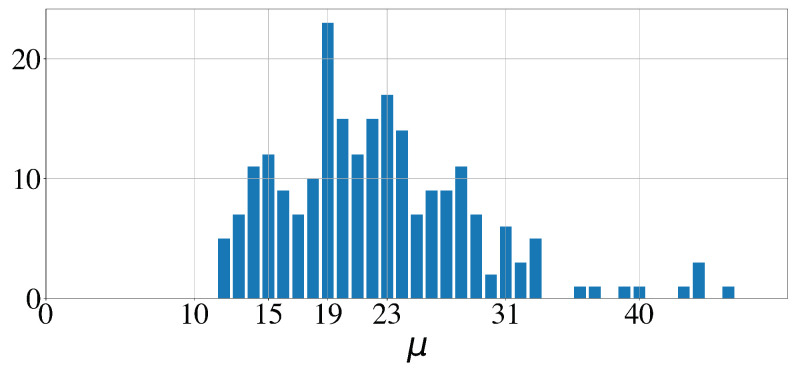
Histogram of significant periodicities identified in 579 sleep bouts, 5 min or longer, by the GMPDA.

Out of the 579 sleep bouts and out of the 100 whole night time series, 183 (31.6%) and 75, respectively, had a loss below 0.75. The corresponding histograms of the significant periodicities extracted from the signals by the GMPDA are shown in Figure 16 and  Figure 17. In both figures, the expected peak in periodicities is around 20. Another minor, rather unexpected, peak is at 15. Significant periodicities ranged from 10 to 33 seconds (except two bouts with a periodicity of 49 and 192 s). Periodicities around 20, i.e., μ∈[17,18,19,20], were present in 95 bouts (out of 183) from 77 subjects (out of 100). Periodicities around 15, i.e., μ∈[12,13,14], were present in 30 bouts from 18 subjects.

Although the minimal periodicity and noise range were set to 5, μ=12 was the smallest periodicity identified by the algorithm for significant bouts.

## 6. Conclusions

In this paper, we developed the Gaussian Mixture Periodicity Algorithm (GMPDA) to address the challenge of detecting overlapping periodicities in noisy data. The GMPDA is based on a novel generative model scheme that explicitly accounts for both a Clock Model and a Random Walk Model. The Clock Model describes periodic behavior in systems where variances do not change over time due to a governing pacemaker, such as scheduled or seasonal behavior like traffic patterns or migration patterns. In contrast, the Random Walk Model describes systems where variances increase over time, making distant temporal predictions difficult or impossible, such as biological behaviors like footsteps or gene expression where events depend only on the interval to the last event.

The primary entry point for the GMPDA is the empirical histogram of all forward-order inter-event intervals. This histogram contains information about the underlying prime periodicities, interaction noise between events associated with different periodicities, and false positive noise. We approximate the overall noise using an explicit formulation under the assumption that the noise is uniformly distributed. This approximation accounts for all interaction intervals, whose lengths are limited by a user-defined parameter in the GMPDA. After subtracting this noise, the GMPDA hierarchically extracts multiple overlapping periodicities by minimizing the loss, defined as the absolute difference between the parametrized histogram obtained by the generative scheme and the empirical histogram.

The GMPDA is implemented in a computationally efficient manner and is available as open-source software on https://github.com/nnaisense/gmpda (accessed on 18 July 2024). We have demonstrated its performance on a set of test cases, including scenarios with up to three overlapping periodicities, different values for Gaussian noise, and varying number of events. For the Random Walk Model, the GMPDA outperformed the FFT and autocorrelation-based approaches as well as the E-periodicity algorithm in identifying true prime periodicities. For the Clock Model, the GMPDA outperformed other algorithms in cases with low variance of intervals.

The GMPDA performed well in the presence of noise with a signal-to-noise ratio of 1:1 and performed adequately up to a ratio of 1:2, given an appropriate number of events. This appropriate number of observed events depends on the signal-to-noise ratio, but more than 30 actual periodic events are generally required for the GMPDA to identify any periodicity.

Finally, we applied the GMPDA to extract significant periods in real data, focusing on leg movements during sleep. The main results here were (i) that the GMPDA was able to identify the expected periodicities around 20 s, (ii) we introduced a procedure to identify a data set-dependent reference loss (of 0.75) to distinguish significant from spurious periodicities, and (iii) our results suggest that there is a minimal number of events (30) required for the GMPDA to perform periodicity detection successfully in biomedical data.

The GMPDA has demonstrated robust performance in detecting periodicity within the framework of the Clock and Random Walk Models. These models are effective for a broad range of scenarios; however, we acknowledge that this focus introduces a limitation in environments where these models may not adequately capture the underlying dynamics of the signal, such as in certain biological systems or financial time series. Nevertheless, the general nature of the generative framework and the formulation of the GMPDA allows for alternative statistical parametrization for the event data. An extension could involve modeling events as a Poisson process, which for multiple periodic generative functions could be modeled as a sum of scaled probability density functions. Additionally, the GMPDA could be extended to periodicity extraction in non-stationary event time series. One approach could involve dividing the time series into locally stationary segments and using a bottom–up segmentation strategy to estimate optimal switching points and prime periodicities for each segment. Another approach could incorporate a Monte Carlo-based particle approach for adaptive periodicity detection as presented in [18]. These extensions remain for future work.

## Figures and Tables

**Figure A1:**
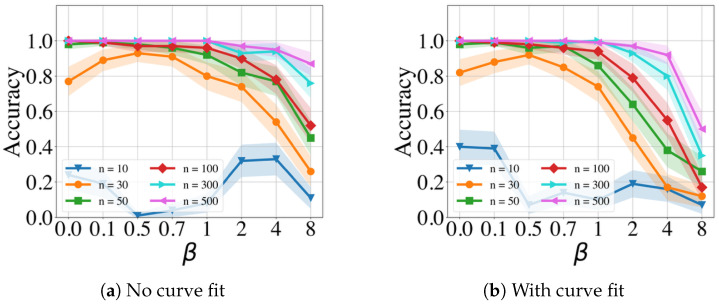
Random Walk Model performance with respect to β, for σ=log(μ) and |μ|=1.

**Figure A2:**
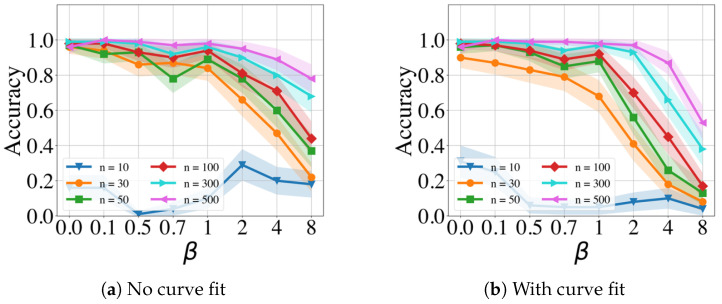
Clock Model performance with respect to β, for σ=log(μ) and |μ|=1.

**Figure A3:**
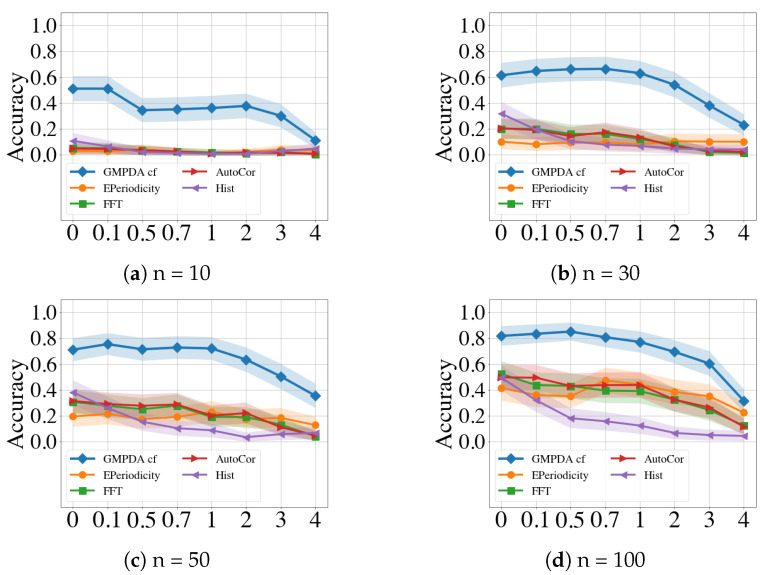

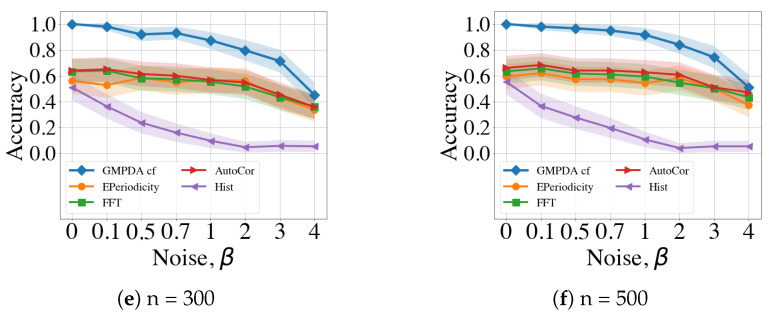
Random Walk Model performance with respect to β, averaged over σ, |μ|=1.

**Figure A4:**
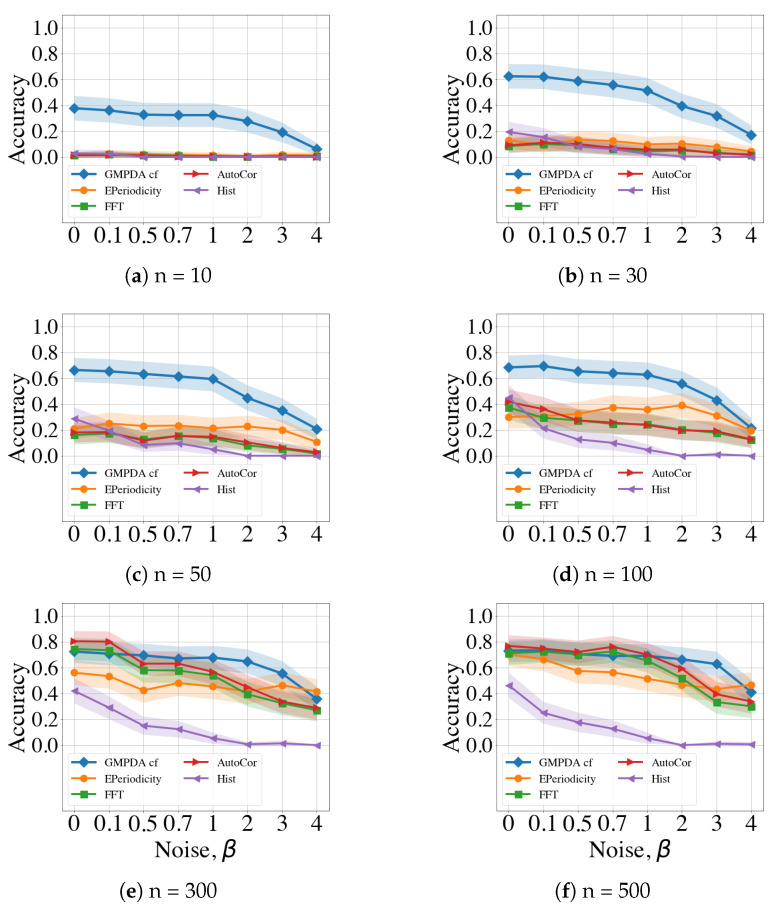
Clock Model performance with respect to β, averaged over σ, |μ|=1.

**Figure A5:**
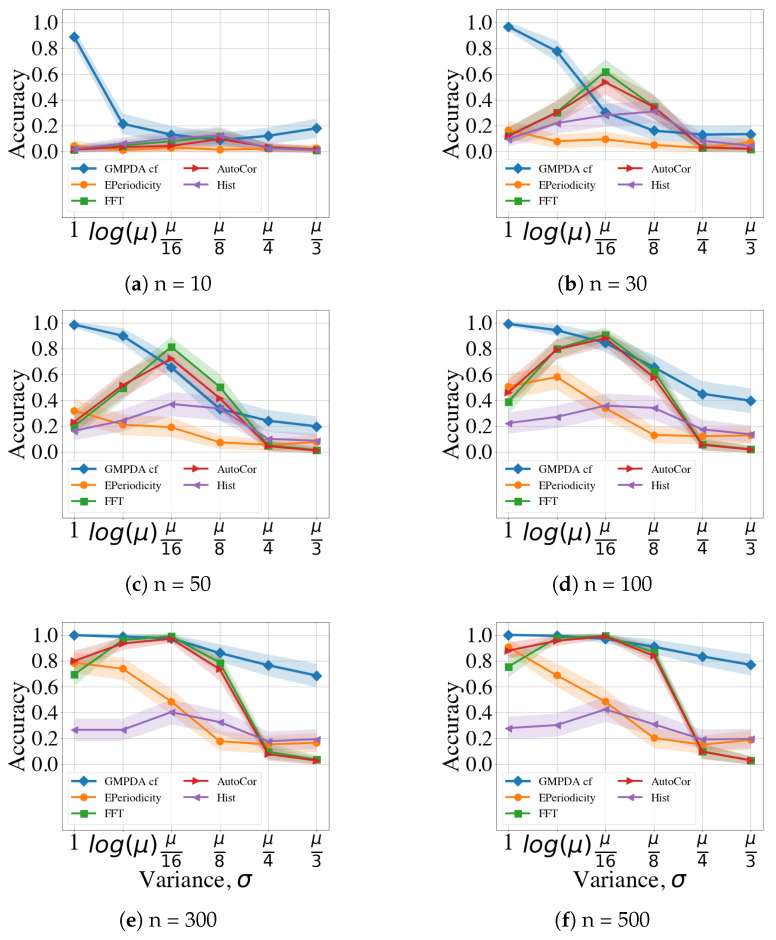
Random Walk Model performance with respect to σ, averaged over noise, |μ|=1.

**Figure A6:**
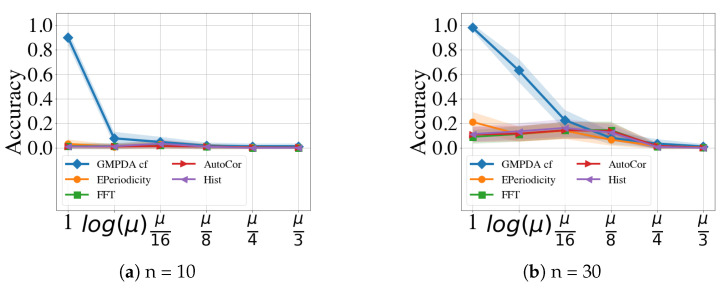

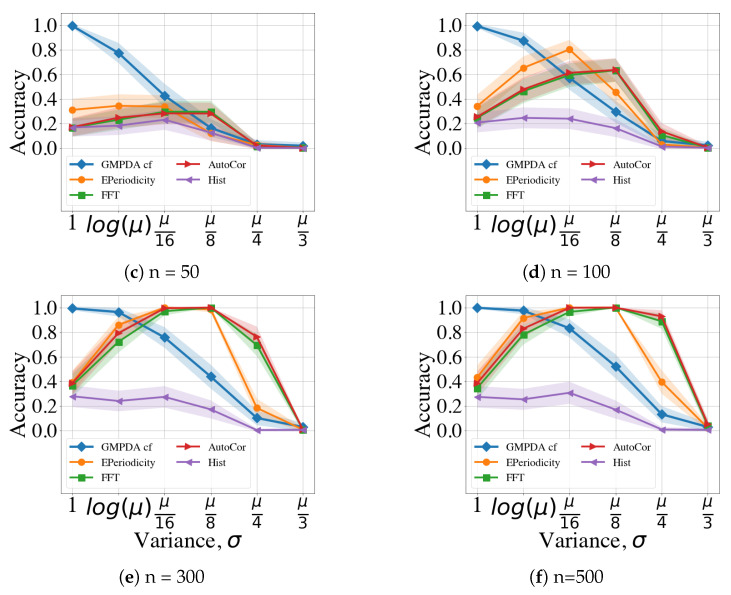
Clock Model performance with respect to σ, averaged over noise, |μ|=1.

## Data Availability

The original data taken from the MrOS Sleep Study are openly available at the National Sleep Research Resource (NSRR) at https://doi.org/10.25822/kc27-0425 (accessed on 17 July 2014).

## Abbreviations

    The following abbreviations are used in this manuscript:

ACF	Autocorrelation Function
FFT	Fast Fourier Transform
GMPDA	Gaussian Mixture Periodicity Detection Algorithm
GMPDA cf	GMPDA with curve fitting
ts	time series

## Appendix A. GMPDA

In this section, we outline in more detail the steps involved in the GMPDA.

### Appendix A.1. Approximation of |ζ(μ)|

In Equation (16), we estimate the number of interaction intervals as a decreasing linear function $E\left[\hat{\zeta }\left(\mu \right)\right]=z\left(1-\frac{\mu }{N_{T}}\right)$. An approximation of *z* is required, as the information about the amount of noise and the number of true periodicities is unknown a priori. Here, we propose the following approximation:

(A1) $$\hat{z}=\frac{1}{z_{min}}\sum_{i=1}^{z_{min}}D\left(\mu \right)\left(i\right).$$

This approximation follows the idea that first, *z* should be close to the maximal value of $E[\hat{\zeta }\left(\mu \right)]$ and that second, all non-zero contributions in D(μ) for $\mu <\underset{\mu }{\mathrm{argmin}}\mu^{*}$ are due to the interaction intervals, asymptotically distributed for an increasing number of events and/or increasing number of involved prime periodicities. For the GMPDA, we set default $z_{min}=L_{min}-1$. Thus, in the algorithm, the length of the interaction intervals is limited either by the minimal expected periodicity, or it can be adjusted by the user.

We verified this approximation empirically on a set of 50,000 test cases with and without noise and a priori known two randomly chosen prime periodicities $\mu {*}_{1/2}\in \left[10,60\right]$: $E[\hat{\zeta }\left(\mu \right)]$ with $\hat{z}$ provided on average a good linear fit, since the distribution of the mean errors between $E[\hat{\zeta }\left(\mu \right)]$ and E[ζ(μ)] was centered at zero and was approximately normal.

However, it must be stressed that the assumption of an uniform distribution of interaction intervals between the periodic and the noise events may be unrealistic in real life data sets, and there is currently no alternative available for estimating zeta from the observed data. This remains an area of improvement for the GMPDA.

### Appendix A.2. Candidate Period Identification

The proposed algorithm hierarchically extracts a set of candidate periodicities, which can explain D(μ) using an integral convolution approach. The method works by iteratively selecting periodicities, which explain many of the observed intervals, and then subtracting the integer multiple intervals which can be explained by these periodicities.

The algorithm takes as input some guess $\hat{\sigma }$, a range in which to search for periodicities $\left\{L_{min},L_{max}\right\}$, a number of hierarchical periodicity extraction steps max_iterations, and a maximal number of periodicities to extract at each hierarchical iteration, max_candidates.

Recall that D(μ) counts the number of times a given interval μ appears between any two events in the time series

(A2) $$D\left(\mu \right)=\sum_{i,m>0}1_{s_{i+m}-s_{i}=\mu }.$$

One tempting method would be to select ${\mathrm{argmax}}_{\mu }D\left(\mu \right)$ as the first prime period ${\hat{\mu }}_{1}$. However, for small or noisy real-world data, argmax may not be the prime period.

We introduce the notion of an *integral convolution*, in which we integrate around a fixed μ to capture how much of the observed intervals in D(μ) are explainable by that particular “mean” periodicity, which also acts to smooth D(μ). We therefore define a function $\tau (\hat{\mu },\hat{\sigma },D\left(\mu \right))$, which will act as a symmetric convolution kernel across D(μ), centered at the candidate means $\hat{\mu }$. This function provides a point-wise estimate of the explained data for a given μ in D(μ):

(A3) $$\tau \left(\hat{\mu },\hat{\sigma },D\left(\mu \right)\right)=\int_{\hat{\mu }-\left(1.96*\hat{\sigma }\right)}^{\hat{\mu }+\left(1.96*\hat{\sigma }\right)}D\left(\mu \right)\partial \mu .$$

Because we do not want to calculate the full loss function (32) at this stage, due to computational expense, we approximate our loss function with the function $\tau (\hat{\mu },\hat{\sigma },D\left(\mu \right))$ for the Clock Model:

(A4) $$E\left({\hat{\mu }}_{p}\right)=\frac{{\hat{\mu }}_{p}}{N_{T}}\cdot \left[\sum_{i=1}^{\frac{N_{T}}{{\hat{\mu }}_{p}}}\tau \left(i\cdot {\hat{\mu }}_{p},\hat{\sigma },D\left(\mu \right)\right)\right],$$

and for the Random Walk:

(A5) $$E\left({\hat{\mu }}_{p}\right)=\frac{{\hat{\mu }}_{p}}{N_{T}}\cdot \left[\sum_{i=1}^{\frac{N_{T}}{{\hat{\mu }}_{p}}}\tau \left(i\cdot {\hat{\mu }}_{p},i\cdot \hat{\sigma },D\left(\mu \right)\right)\right].$$

Functions (A4) and (A5) approximate, for each candidate prime period, how much of the data can be explained by this periodicity in some confidence interval about $\mu_{p}$. If a periodicity is present and is persistent through the time series, integer multiples of the periodicity ${\hat{\mu }}_{p}$ will also frequently appear in (A4) and (A5); we can use this information to select the periodicity which explains the most data.

Once the first periodicity ${\hat{\mu }}_{1}$ has been identified, we remove the intervals found in D(μ), which can be explained by ${\hat{\mu }}_{1}$, i.e., integer multiples of ${\hat{\mu }}_{1}$. We realize this by setting D(μ) to zero for $\mu \in [i\cdot {\hat{\mu }}_{1}+\hat{\sigma },i\cdot {\hat{\mu }}_{1}-\hat{\sigma }],\;i=1,\cdots$, and recompute τ(μ) from D(μ) missing these intervals.

The GMPDA performs reasonably well at identifying the true periods $\mu^{*}$ as $\hat{\mu }$ without the use of the loss function or adjustments to $\hat{\sigma }$. But without a measure of relative goodness of this estimates, we have no stopping criteria for finding multiple periodicities. Instead, we repeat this procedure for max_iterations iterations.

Once we have initialized (hierarchically) a set of candidate prime periods ${\hat{\mu }}^{init}$ using this “fast” method, we compute a better estimate of the variance and loss using methods which are elaborated in the following sections.

### Appendix A.3. Non-Linear Least Squares Fitting for σ ^

We can improve our guess of the variance $\hat{\sigma }$ by formulating a non-linear least squares curve fitting optimization problem, in which our set of parameters comprises those of a Gaussian PDF. That is, here we consider D(μ), which can be modeled as the sum of Gaussian PDFs, and a set of candidate means $\hat{\mu }$ for those Gaussian PDFs. For a fixed set ${\hat{\mu }}_{p}$, we initialize guesses for ${\hat{\sigma }}_{p}$, for p=1,⋯,P, and deploy the Trust Region Reflective algorithm to obtain an update for the guesses of ${\hat{\sigma }}_{p}$. It is implemented with curve_fit() from Scipy’s optimization package.

### Appendix A.4. Selecting True Parameters: Loss Function

The parameter estimates ${\hat{\mu }}^{init}$, *M*, $\hat{\sigma }$ are assessed with respect to D(μ)—the observed intervals between events in the time series—using a loss function. This loss function describes the proportion of intervals in the data, which can be explained with (i) the parametrized “generative function” $G_{M}\left(\hat{\mu },\hat{\sigma }\right)$ implicated by the estimates, which is asymptotically the same as the expectation of D(μ) if $\hat{\mu }=\mu^{*}$ and $\hat{\sigma }=\sigma^{*}$ and (ii) the noise approximation.

Computing the loss function (32) is expensive because in the case of the Random Walk Model, $G_{RW}$ requires computation at increasingly large intervals since the variance terms grow linearly, and thus the area covered with some density by a single Gaussian distribution grows at the same rate. Thus, we only want to compute $G_{M}$ for a few very probable periodicities (the set ${\hat{\mu }}^{init}$ computed in the fast algorithm), using the optimal variance guesses $\hat{\sigma }$, and only across a limited range of intervals specified by loss_length chosen a priori.

We also adjust the scaling factor of the generative function to account for sections of the time series which may not have any events, for instance, missing values or large intervals of the time series with no observations. This concerns the scaling factor $c_{p}$, for p=1,⋯,P, of $G_{M}\left(\hat{\mu },\hat{\sigma }\right)$ for the Clock Model and Random Walk Model in Equations (28) and (29), respectively. The adjusted scaling factor is

(A6) $$c_{p}=\frac{N_{T}\left({\hat{\mu }}_{p}\right)}{{\hat{\mu }}_{p}}-\left(m-1\right),$$

where $N_{T}\left({\hat{\mu }}_{p}\right)$ is the sum of intervals which are smaller than ${\hat{\mu }}_{p}+\left({\hat{\sigma }}_{p}\cdot 2\right)$. This correction ensures that we only count “possible appearances” in the time series on sections which actually have events. Without this scaling factor, missing values would bias our results towards higher frequencies, and the scaling factor would be far too large for lower frequencies which may appear in the time series but with intervals of no events.

Our final loss function will therefore be constructed using D(μ) computed from the real data, $E[\hat{\zeta }\left(\mu \right)]$, $G_{M}\left({\hat{\mu }}_{init},\hat{\sigma }\right)$, and one additional parameter, loss_length. This parameter manages high variance at high integer multiples and decreases the computational complexity. In the Random Walk Model, for high integer multiples of a periodicity, the implied Gaussian distributions of intervals begin to have large tails and the distribution density mean decreases. Meanwhile for the Clock Model, estimating many integer multiples is not actually necessary to compute the true periods. Therefore, the loss we compute in the algorithm is:

(A7) $$\hat{L}=\sum_{\mu =0}^{loss\_length}\left|D\left(\mu \right)-\hat{\zeta }\left(\mu \right)-G_{M}\left(\hat{\mu }\right).\hat{\sigma }\right|,$$

Within the algorithm, we compute $G_{M}$ for all combinations of the set ${\hat{\mu }}^{init}$ up to order max_combi, and select our true set of periodicities as that which minimizes (A7). Please note that the number of true periodicities max_combi is not known a priori; the optimal value of max_combi will minimize the loss. However, in real applications, we might have situations where there are weak peaks in D(μ) around very large μ due to noise or the influence of large/slow interactions intervals. Adding these to the set of prime periodicities will decrease the loss, but will not contribute to the identification of intrinsic periodicities. To account for this, the GMPDA provides the possibility to control the magnitude of the loss decrease by a parameter loss_decrease_tol, with the loss being typically of magnitude one and lower; see Section 5. That is, setting this tolerance parameter to a very low number, e.g., loss_change_tol=0.001, will result in including more periodicities (that might be due to noise), while a larger number, e.g., loss_change_tol=0.1, will be more conservative.

## Appendix B. Performance

### Appendix B.1. |μ|=1

Here, the performance of the GMPDA with respect to noise with curve fitting and without curve fitting is presented.

The Random Walk Model model exhibits a decay in performance with an increasing noise for n>30. For n=30, the performance increases until β=0.5, as the noise, to a certain extent, is acceptable due to the definition of the variance for the Random Walk Model 5.

The Clock Model exhibits for n>10 a decay in performance with an increase in β. For both models, the case n=10 performs insufficiently, indicating that the number of events must be definitely higher than ten.

### Appendix B.2. Comparison to Alternative Methods with Respect to Noise β

Here, we compare the performance of the GMPDA and of the alternative methods regarding an increase in noise. In the following figures, the accuracy of all the involved methods is plotted for |μ=1|, different number of events *n*, while it is averaged over all considered values of variance σ. The GMPDA with curve fitting consistently outperformed the alternative methods for different levels of noise for the Random Walk Model (Figure A3) for up to 500 events, and up to 100 events for the Clock Model (Figure A4).

### Appendix B.3. Comparison to Alternative Methods with Respect to Variance σ

Figure A5 and Figure A6 compare the performance of the GMPDA and the alternative methods for increasing levels of variance. In the following figures, the accuracy of all the involved methods is plotted for |μ=1| and different number of events *n*, while it is averaged over all considered values of noise β.

### Appendix B.4. Sensitivity Analysis

We summarize the differences in sensitivity to critical simulation parameters across the different algorithms in Table A1. Based on the simulation results obtained in Section 4, we used generalized linear mixed models with number of periods |μ|=1,2,3 nested within trials (*n* = 38,400), with the response being the accurate detection of a single periodicity (coded as 1 if the estimate is within an intervals around the true value ±σ) and the independent factors being as follows:

- Number of events n=10,30,50,100,300,500;
- Number of periods |μ|=1,2,3;
- Variance $\sigma =1,\log\left(\mu \right),\frac{\mu }{p},\;\mathrm{with}\;p=3,8,16$;
- Noise β=0,0.1,0.5,0.7,1,2,4,8.

Mixed logistic models were computed separately for the Clock Model and the Random Walk Model and each algorithm. Table A1 lists the ANOVA type II sum of squares (SoS), i.e., the SoS of each main effect after the introduction of all other main effects. While the SoS are not directly comparable between models, their relative contribution is, and suggests that for the Random Walk Model, the number of events had a major effect in all algorithms except the FFT histogram algorithm. The number of periods had a small to moderate effect, except for the E-periodicity, where it did not play a role. Both E-periodicity and the GMPDA with curve fitting were very sensitive to the noise level, and across algorithms, variations in sigma had one of the strongest effects on accuracy, again with the exception of the E-periodicity algorithm.

The results for the Clock Model were largely similar with some notable exceptions. Compared to the Random Walk Models, the E-periodicity algorithm was considerably less sensitive to variations in noise but more sensitive to variations in variance. Overall, the three algorithms E-periodicity, FFT, and FFT autocorrelation showed a similar pattern, with the number of events having the strongest influence, sigma being the second strongest, and the noise and number of periods having only relatively minor effects. For the two GMPDAs, the strongest effect was seen for sigma.

Across all models and algorithms, both sigma and the number events emerged as the strongest determinants of periodicity detection accuracy.

**Table A1:** Differences in sensitivity to critical simulation parameters across the different algorithms. The table lists the ANOVA type II sums, with larger numbers signifying larger sensitivity to the respective parameter.

	Number of Events	Number of Periods	Noise	Sigma Ratio
Df	5	2	7	5
**Random Walk Models**				
GMPDA with curve fitting	5110	3746	5096	4756
GMPDA w/o curve fitting	3349	1821	1291	7830
E-periodicity	3218	10	5221	438
FFT	5519	1420	1359	6687
FFT Autocorrelation	5506	1252	3058	6013
FFT Histogram	619	1159	958	2994
**Clock Models**				
GMPDA with curve fitting	2714	2377	3186	7399
GMPDA w/o curve fitting	2089	454	1210	6806
E-periodicity	7659	280	651	4707
FFT	8520	157	1522	5216
FFT Autocorrelation	8480	87	1283	5689
FFT Histogram	438	211	2829	1035

### Appendix B.5. Real Application: Loss

This section shows the GMPDA loss obtained with and without curve fitting for the MROS data set. Figure A7 shows the loss for 100 recordings in the left panel and the loss comparison for all single bouts in the right panel.
